# A comparative study of germline *BRCA1* and *BRCA2* mutation screening methods in use in 20 European clinical diagnostic laboratories

**DOI:** 10.1038/bjc.2017.223

**Published:** 2017-07-27

**Authors:** Gillian Ellison, Andrew Wallace, Alexander Kohlmann, Simon Patton

**Affiliations:** 1 AstraZeneca, Personalised Healthcare and Biomarkers, Alderley Park, Macclesfield SK10 4TG, UK; 2 Genomic Diagnostics Laboratory, Manchester Centre for Genomic Medicine, St Mary’s Hospital, Manchester M13 9WL, UK; 3 European Molecular Genetics Quality Network, Manchester Centre for Genomic Medicine, St Mary’s Hospital, Manchester M13 9WL, UK

**Keywords:** BRCA, NGS, method, ovarian, cancer

## Abstract

**Background:**

Thousands of clinically relevant variations in *BRCA1* and *BRCA2* have been discovered and this poses a significant challenge with respect to the accurate detection, analysis turn-around time, characterisation and interpretation of these sequence variants.

**Methods:**

We evaluated the performance of different *BRCA1/2* gene testing practices in routine diagnostic use in 20 European laboratories, with a focus on next-generation sequencing-based strategies as this is the technical approach implemented by or under adoption by most European clinical laboratories. Participant laboratories, selected on expertise and diagnostic service quality, tested 10 identical DNA samples containing a range of challenging pathogenic variants.

**Results:**

A small number of errors in the detection of pathogenic and significant variants were identified (2.6% diagnostic error rate). There was a high degree of concordance (>97%) across all laboratories for all variants detected. No systematic technical flaw was identified in the strategies employed across the participating laboratories.

**Conclusions:**

The discrepancies identified are most likely due to human error or the way the methodology has been implemented locally, for example, next-generation sequencing bioinformatics pipelines, rather than technical limitations of the methods. The choice of *BRCA1/2* testing method will therefore depend on multiple factors including required throughput and turn-around times, access to equipment, expertise and budget.

Mutations in the *BRCA1* and *BRCA2* genes lead to an increased risk of developing breast or ovarian cancer. Women who are heterozygous for a *BRCA1* or *BRCA2* pathogenic variant have up to an 80% risk of developing breast cancer by age 90; and an ovarian cancer risk of about 55% with *BRCA1* mutations and 25% with *BRCA2* mutations (Malander *et al*, 2004; Majdak *et al*, 2005; Alsop *et al*, 2012; Dann *et al*, 2012). Thousands of clinically relevant variations in *BRCA1* and *BRCA2* have been discovered to date, and these are distributed widely throughout the entire coding regions and intron–exon boundaries (Casey, 1997). This poses a significant challenge with respect to the accurate variant detection, analysis turn-around time, characterisation and interpretation of *BRCA1* and *BRCA2* sequence variants.

*BRCA1/2* mutation screening is provided by a large number of clinical diagnostic laboratories worldwide using various different analytical methods and technology platforms. Laboratory practice is changing rapidly due to increased demand for testing (Palma *et al*, 2008; Public Policy Institute A, 2015), the advent of treatment-focused genetic testing (Ledermann *et al*, 2014) and the rapid uptake of next-generation sequencing (NGS) technologies (Idris *et al*, 2013; Trujillano *et al*, 2015). It is critically important that the result of the *BRCA1/2* test is accurate, as significant clinical decisions are being made based on the results, such as prophylactic surgery to reduce the risk of cancer development. Information on *BRCA1/2* mutation status is now being used to inform treatment decisions such as the use of platinum-based chemotherapy (Christie *et al*, 2014) and to guide the use of PARP inhibitors (Ledermann *et al*, 2014).

In this methodological assessment, we evaluated the performance of different *BRCA1/2* gene testing methods in current routine clinical practice among European Molecular Genetics Quality Network (EMQN) members to determine how they compare at detecting clinically relevant *BRCA1/2* variants. As a first step (Part A), the most common actively used methods for germline *BRCA1/2* mutation analysis were determined through a questionnaire sent to active EMQN member laboratories participating in External Quality Assessment (EQA) for BRCA testing.

The study chose to focus on NGS and associated bioinformatics pipelines since this is the technical approach most labs are now using for *BRCA1/2* testing. Therefore a subset of 20 laboratories representing 11 European countries were selected for further analytical evaluation in this study (Part B). Fifteen of these laboratories were using NGS, while the remaining five were using more traditional testing methodologies, that is, Sanger sequencing, denaturing high-performance liquid chromatography (dHPLC) and high-resolution melting HRM. Where possible, each method was undertaken by more than one participating laboratory to provide a comparison of the method rather than the individual laboratory. All of these laboratories were required to undertake *BRCA1/2* testing on a range of highly characterised DNA samples with known *BRCA1/2* genotypes, including a wide range of commonly encountered *BRCA1* and *BRCA2* variants encompassing a range of single-base substitutions, giving rise to missense, nonsense and splice-site mutations, insertion/deletion frameshift mutations, single exon deletions and large genomic rearrangements (LGR).

## Materials and methods

### Initial participant laboratory selection (Part A)

In Part A of the study a short survey of current *BRCA1/2* gene screening methods was circulated to 156 members of the EMQN network participating in the 2014 EQA scheme for BRCA testing. The survey was used to establish the current state-of-the-art in *BRCA* testing among the EMQN member laboratories and to inform laboratory selection for Part B of the study.

### Criteria for selection of laboratories for analytical sample investigation (Part B)

Laboratories were selected to participate in Part B of the study based on their responses to the Part A survey. Participation by selected laboratories was voluntary – all agreed to take part. These laboratories had demonstrated a significant diagnostic *BRCA* testing caseload per annum (>300 germline *BRCA1/2* mutation analyses), conducted all testing in their own laboratory (no outsourcing or subcontracting of their testing process) and had demonstrated evidence of successful participation in three successive years in a recognised External Quality Assurance (EQA) scheme (e.g., EMQN, UK National External Quality Assessment Scheme (UK NEQAS) for Molecular Genetics, or College of American Pathology (CAP)). Where possible, we also selected laboratories which were accredited to the ISO15189 medical laboratory standard as this gave additional assurances and confidence that the lab was performing high-quality testing.

Laboratories using NGS as their primary mutation detection assay were sent an additional survey to establish the details of their bioinformatics analysis pipelines used to identify mutations, and the minimum standards of sequencing coverage that were applied to their diagnostic analysis.

### Study procedure (Part C)

In total, 10 DNA samples extracted from lymphoblastoid cell lines as reference material (Table 1) were distributed to the 20 participating laboratories. These samples included eight specimens that contained a diverse range of *BRCA1/2* mutation types (LGRs, missense, nonsense, splice and indels) while the remaining two contained no *BRCA*1/2 mutations. A total of seven pathogenic mutations and two variants of unknown significance (VUS) were supplied (see Table 1 for details). All participating laboratories were given two calendar months to complete their analyses using their standard clinical diagnostic methodology and were asked to report any clinically significant findings (i.e., the significant differences from the cDNA reference sequences *BRCA1* NM_007294.3 and *BRCA2* NM_000059.3) in addition to making available their ‘raw’ data if required. Large genomic rearrangements analysis of the samples was not mandated as a precondition for participation in the study. Laboratories were requested to analyse samples using their standard methodology with the intention that those using an NGS approach to detect LGRs would do so. Given this, some laboratories that used an NGS approach unsuitable for LGR analysis did not analyse the samples for LGRs although analysis with an independent method, for example, multiplex ligation-dependant probe amplification (MLPA) would be their standard practice.

## Results

### Laboratory distribution and *BRCA1/2* screening methods

Eighty-nine (57%) laboratories responded to the survey. Twenty laboratories distributed across 11 EU countries were selected from the 89 applicants. In detail, six labs were from the UK, two labs each from France, Belgium, Germany and Italy, and one lab from Spain, Denmark, Greece, The Netherlands, Czech Republic and Austria, respectively. Fifteen of the participating labs used NGS as their primary *BRCA1/2* mutation detection strategy, three used Sanger sequencing and two used a pre-screening strategy (high resolution melt (HRM) plus dHPLC analysis, or HRM analysis alone), followed by Sanger sequencing to define any variants identified via the pre-screening process. The NGS laboratories used a variety of different instruments and sequencing chemistries as well as sample preparation and library enrichment methods. The strategies used by the laboratories are described in more detail in Table 2.

Thirteen out of 20 (65%) of the participating laboratories analysed all the samples for LGRs. Eight of these used commercial MLPA probe sets while another five used NGS (with hybridisation enrichment) to directly detect LGRs. Three of the laboratories using MLPA only tested for the presence of *BRCA1* deletions and duplications.

A wide range of bioinformatics approaches and tools were used with little consensus between the laboratories on the minimum and mean coverage cut-off parameters that were applied to NGS data. The target read depth across the region of interest varied from 30-fold to 500-fold coverage and the minimum acceptable read depth ranged from 10 × to 100 ×.

### Concordance analysis of pathogenic *BRCA1* and *BRCA2* variants

All participant laboratories managed to complete their testing procedures for all of the samples provided. Given that there were nine pathogenic mutations/VUSs within the 10 samples, and allowing for the seven laboratories that did not analyse the samples for LGRs and three laboratories that only analysed for LGRs in *BRCA1*, there were 156 opportunities in total to detect pathogenic mutations/VUSs across the 20 laboratories (Table 3).

Importantly, no false-positive mutation/VUS calls were made across the 20 laboratories, resulting in 100% specificity of the mutation analyses. However, four pathogenic mutations were missed by three laboratories. The overall sensitivity of mutation/VUS detection across the laboratories was therefore 97.4% (152 out of 156).

In detail, laboratory 11 failed to identify two mutations; a 40 bp deletion in *BRCA1* c.1175_1214del40, p.(Leu392Glnfs*5) and a splice-site mutation in *BRCA2* c.7617+1G>T. Failure to identify these mutations is likely to be due to the specific bioinformatics analysis used in this centre, as laboratory 12 used exactly the same NGS technology and correctly identified both mutations.

One laboratory (laboratory 14) using the MRC-Holland MLPA *BRCA1* P002-C3 kit missed a LGR. This same kit was also used by eight other laboratories to successfully detect *BRCA1* LGRs in the study. Independent re-analysis of the MLPA data from this laboratory showed no evidence of the expected exon 3–8 duplication. After discussion with the laboratory we concluded that the most likely reason for failing to identify the mutation was a sample swap error during their MLPA analysis, as NGS analysis by the same laboratory did identify the rare *BRCA1* c.4393A>C, p.(Ile1465Leu) VUS, which was also present in this sample.

Laboratory 16, using an HRM pre-screening strategy, failed to identify the *BRCA2* nonsense mutation c.10G>T; p.(Gly4*) in one sample. Root cause analysis by the laboratory identified that the mutation was missed because the HRM profile was very similar to a known polymorphic variant within the amplicon containing the mutation. The laboratory did not undertake any confirmatory testing to directly identify the DNA change causing the aberrant melting profile.

Overall, the small number of errors in the detection of pathogenic and significant variants did not reveal any particular vulnerability in any of the mutation detection strategies employed across all participating laboratories.

### Concordance analysis of all *BRCA1/2* variants

In order to increase the power of the study to discriminate between the different analysis methods, we analysed the neutral (polymorphic) variants that were present in the test samples. Each participating laboratory was asked to report all the differences from the cDNA reference sequences (*BRCA1* NM_007294.3 and *BRCA2* NM_000059.3) that they identified in the sample set. We supplied a standard data sheet for completion in HGVS compliant format and also requested the zygosity of each variant detected. Since each laboratory had analysed intronic and non-coding regions of the samples to different extents we restricted the comparison to coding sequence variants only.

Across the 10 samples analysed there were 40 *BRCA1* and 40 *BRCA2* neutral coding region variants where the sequence in the test sample differed from the reference sequence. There were 20 unique single-nucleotide variants variants in total, 11 in *BRCA1* and 9 in *BRCA2* (Table 3). The analysis of the variant data was split into laboratories carrying out NGS analysis and those using Sanger sequencing. No analysis was done on the data from laboratories 15 and 16 as they were using a pre-screening only strategy with methods that do not specify the precise DNA sequence alteration. Notably, analysis of the pattern of variant genotypes reported by some laboratories showed that they had used an alternative reference sequence for *BRCA2* (U43746.1) to analyse their data rather than the reference sequence, NM_000059.3, requested by the authors. Where the pattern of genotypes reported for a laboratory matched the use of U43746.1, the reported genotypes were scored against this reference sequence and the variances from expected were not counted as an error.

In total, of the 18 laboratories using a DNA sequencing technology (NGS or Sanger), 10 (55%) made no errors in the reported variant genotypes. Of the remaining 8 laboratories a total of 27 discordances from the expected genotype were reported. The discordances were broken down into four categories as follows: (i) Failure to detect a variant that deviates from the reference sequence (false negative) – 10 occurrences; (ii) Detection of a variant from the reference that is not present (false positive) – 6 occurrences; (iii) Zygosity incorrectly determined for a variant – 4 occurrences; (iv) Typographical error – 7 occurrences. Errors were categorised as typographical where a novel change was reported that shared at least three digits with a genuine variant that was not reported, for example, *BRCA1* c.4873A>G heterozygous reported instead of *BRCA1* c.4837A>G heterozygous.

Overall, the accuracy of genotyping the 1440 variants that were present in the 10 samples was 1413 out of 1440 (98.1%). This is close to the accuracy estimate of 97.4% from the 156 clinically relevant variants that were tested in the main phase of the study. The distribution of discordances across the sequencing laboratories is given in Table 4.

Interestingly both laboratories using an NGS screening technology that made errors in the reporting of clinically relevant variants also made errors in the reporting of all other variants (laboratories 11 and 14). There did not appear to be any overall pattern between the variants that were not detected between labs or samples, with errors seeming to be randomly distributed.

Interestingly, laboratory 4 conducted a more detailed analysis of the three errors that it had made in this part of the study. This laboratory had used Illumina sequencing chemistry coupled with a custom long PCR amplicon library preparation approach. All three errors (two for incorrect zygosity, and one for a variant not identified) were in the same sample (sample 6) and occurred in the same long PCR amplicon. One of the PCR primers for this amplicon overlaps the pathogenic *BRCA2* deletion present in this sample (*BRCA2* c.6842-?_7007+?del). Consequently, the presence of the large deletion leads to analytical interference preventing amplification of one of the *BRCA2* alleles. Any mutation detection strategy dependant on PCR enrichment is prone to interference arising from such variants in the primer hybridisation sequences. Consequently, PCR primers for clinical assays should be carefully designed to avoid common variants that may cause interference in target amplification.

## Discussion

In this study we have observed that various methods used by experienced clinical laboratories performed well on a range of challenging *BRCA1/2* mutations. No single NGS method and associated bioinformatics pipeline was demonstrated to be superior, and were equally as capable of detecting the range of significant variants as more established methods. The results were shared between the study participants and have subsequently been used to improve practice in EQA schemes for BRCA testing and NGS.

The discrepancies in the identification of pathogenic and significant variants are most likely due to human error or the way the methodology has been implemented locally, for example, NGS bioinformatics pipelines, rather than technical limitations of the methods. Robust processes to eliminate sample mix-ups are essential and important to ensure high-quality testing. The extended analysis of neutral variants did not reveal any further specific vulnerabilities in the technologies used; however it did confirm the genotyping error rate of NGS analysis in *BRCA1/2* to be in the range of 2–3%. This is consistent with the error rates identified in EQA schemes for *BRCA*1/2 (Mueller *et al*, 2004), (Simon Patton, personal communication) and other inherited genetic disease (Dequeker *et al*, 2001; Seneca *et al*, 2008). The actual error rate in clinical practice is likely to be lower than the 2–3% identified in the study, as laboratories were not required to fully replicate their diagnostic reporting pathway. A significant proportion of the errors in the extended study were clerical, or likely to be, and the error would be expected to be detected during checks undertaken during the clinical reporting process. Consequently an error rate of 2–3% should be viewed as the upper boundary of diagnostic error in these experienced laboratories. Nevertheless the error rate in less experienced laboratories could be higher, as evidenced in EQA schemes for molecular pathology. In molecular testing for somatic mutations, the diagnostic testing process has evolved rapidly, as new treatment-related outcomes to gene alterations have become available (Normanno *et al*, 2013). Consequently, many laboratories have implemented testing strategies without rigorous validation or method verification. External Quality Assessment schemes in this area of diagnostic testing have demonstrated significantly higher error rates (Deans *et al*, 2011; Wong *et al*, 2012; Patton *et al*, 2014) but also improvements over time (Deans *et al*, 2013; Patton *et al*, 2014). It is therefore essential for all laboratories offering *BRCA*1/2 testing to carry out robust assay validation and participate in regular EQA in order to minimise the possibility of errors in diagnostic screening.

In conclusion, It is our understanding that the choice of *BRCA1/2* testing method will therefore depend on multiple factors including required throughput and turn-around times, access to equipment, expertise and budget.

## Figures and Tables

**Table 1:** Reference materials used in the study

	**Confirmed** ***BRCA1/2*** **mutation status**			
**Sample**	***Gene*** **(RefSeq)**	**Mutation/variant 1**	**Mutation/variant 2**	**Comments**
1	*BRCA1* (NM_007294.3)	c.1175_1214del40, p.(Leu392Glnfs*5)	NA	40 bp deletion (frameshift)
2	*BRCA1* (NM_007294.3); *BRCA2* (NM_000059.3)	NA	NA	Control sample–no pathogenic mutations
3	*BRCA2* (NM_000059.3)	c.10G>T; p.(Gly4*)	NA	Point mutation (nonsense)
4	*BRCA1* (NM_007294.3)	c.442-?_547+?del,	NA	LGR: deletion (exon 8)
5	*BRCA2* (NM_000059.3)	c.5909C>A; p.(Ser1970*)	NA	Point mutation (nonsense)
6	*BRCA2* (NM_000059.3)	c.6842-?_7007+?del,	NA	LGR: deletion (exons 12–13)
7	*BRCA2* (NM_000059.3)	c.7617+1G>T; p.?	NA	Point mutation (splice site)
8	*BRCA1* (NM_007294.3)	c.81-?_547+?dup;	c.4393A>C, p.(Ile1465Leu)	LGR: duplication (exons 3–8) and UV
9	*BRCA1* (NM_007294.3)	c.3022A>G; p.(Met1008Val)	NA	UV
10	*BRCA1* (NM_007294.3); *BRCA2* (NM_000059.3)	NA	NA	Control sample–no pathogenic mutations

Abbreviations: LGR=large genomic rearrangements; NA=not applicable; UV=unclassified variant.

**Table 2:** Details on test methodologies applied by participant laboratories

**Laboratory**	**Primary test strategy**	**NGS platform**		**Method**
Lab 1	Sequencing (NGS)	Illumina	HiSeq2500	Hybridisation selection (TruSight Cancer Panel (Illumina))
Lab 2	Sequencing (NGS)	Illumina	NextSeq500	Hybridisation selection (TruSight Cancer Panel (Illumina))
Lab 3	Sequencing (NGS)	Illumina	MiSeq	Long amplicon (Lab Developed Test)
Lab 4	Sequencing (NGS)	Illumina	MiSeq	Long amplicon (Lab Developed Test)
Lab 5	Sequencing (NGS)	Illumina	MiSeq	Short amplicon (*BRCA1/BRCA2/TP53* kit (Fluidigm))
Lab 6	Sequencing (NGS)	Illumina	MiSeq	Hybridisation selection (Lab Developed Test (Haloplex))
Lab 7	Sequencing (NGS)	Illumina	MiSeq	Short amplicon (*BRCA* MASTR Dx (Multiplicom))
Lab 8	Sequencing (NGS)	Illumina	MiSeq	Short amplicon (*BRCA* MASTR Dx (Multiplicom))
Lab 9	Sequencing (NGS)	Illumina	MiSeq	Hybridisation selection (Lab Developed Test)
Lab 10	Sequencing (NGS)	Illumina	MiSeq	Hybridisation selection (SureSelect (Illumina))
Lab 11	Sequencing (NGS)	Life Technologies 1	IonPGM	Short amplicon (Ion AmpliSeq *BRCA1* and *BRCA2* Panel (Life Technologies))
Lab 12	Sequencing (NGS)	Life Technologies	IonPGM	Short amplicon (Ion AmpliSeq *BRCA1* and *BRCA2* Panel (Life Technologies))
Lab 13	Sequencing (NGS)	Life Technologies	IonPGM	Short amplicon (*BRCA* MASTR Dx (Multiplicom))
Lab 14	Sequencing (NGS)	Roche Diagnostics	GS Junior	Short amplicon (*BRCA* MASTR Dx (Multiplicom), Multiplicom *BRCA* HP (Multiplicom))
Lab 15	Sequencing (NGS)	Roche Diagnostics	GS Junior	Short amplicon (*BRCA* MASTR Dx (Multiplicom))
Lab 16	HRM, dHPLC	NA	NA	NA
Lab 17	HRM	NA	NA	NA
	Sequencing (Sanger)			
Lab 18	Sequencing (Sanger)	NA	NA	NA
Lab 19	Sequencing (Sanger)	NA	NA	NA
Lab 20	Sequencing (Sanger)	NA	NA	NA

Abbreviations: dHPLC=denaturing high performance liquid chromatography; HRM=high resolution melting; NA=not applicable; NGS=next-generation sequencing.

**Table 3:** Distribution of 20 unique SNV sequence variants and their zygosity.

	***BRCA1*****(NM_007294.3)**											***BRCA2*****(NM_000059.3)**								
**Sample**	**c.2082C>T**	**c.2311T>C**	**c.2315T>C**	**c.2612C>T**	**c.3113A>G**	**c.3119G>A**	**c.3548A>G**	**c.4308T>C**	**c.4393A>C**	**c.4837A>G**	**c.4956G>A**	**c.1114A>C**	**c.3396A>G**	**c.3807T>C**	**c.4258G>T**	**c.4563A>G**	**c.5744C>T**	**c.5987C>G**	**c.6513G>C**	**c.7242A>G**
1			1									1	1			2			2	
02^a^	2	2		2	2		2	2		2			2		1	2			2	
3	1	1		1	1		1	1		1	1	1		1		2			2	
4	1	1		1	1		1	1		1	1	1		1		2			2	
5													2			2			2	2
6												1	1			2	1	1	2	
7	1	1		1	1		1	1		1			1			2			2	
8						1			1				1	1		2			2	
9													1			2			2	
10^a^	2	2		2	2		2	2		2			2		1	2			2	

Abbreviations: SNV=single-nucleotide variants; 1=heterozygous; 2=homozygous.

a Samples 02 and 10 were replicates of the same sample.

**Table 4:** Distribution of discordant results reported by sequencing laboratories

	**Laboratory**																	
**Error type**	**1**	**2**	**3**	**4**	**5**	**6**	**7**	**8**	**9**	**10**	**11**	**12**	**13**	**14**	**17**	**18**	**19**	**20**
False -ve	0	0	0	1	0	2	1	4	0	0	2	0	0	0	0	0	0	0
False +ve	0	0	0	0	0	0	0	0	0	0	0	0	0	0	6	0	0	0
Incorrect zygosity	0	0	0	2	0	0	0	0	0	0	0	0	0	1	0	0	1	0
Typographical	0	0	0	0	0	1	0	0	0	0	1	0	0	0	0	0	5	0
Total	0	0	0	3	0	3	1	4	0	0	3	0	0	1	6	0	6	0
